# Supplementary material for: A Systemically Administered Humanized Anti-Nav1.7 Antibody with Long-Lasting Analgesic Activity and Preserved Physiological Nociception
Source: Pharmaceutics. 2026 Jun 21;18(6):757. doi: 10.3390/pharmaceutics18060757 (PMC13306241; doi:10.3390/pharmaceutics18060757)
Supplement: Supplementary file 1 [file pharmaceutics-18-00757-s001.zip › pharmaceutics-4321109-supplementary.pdf]

Supplementary Figure

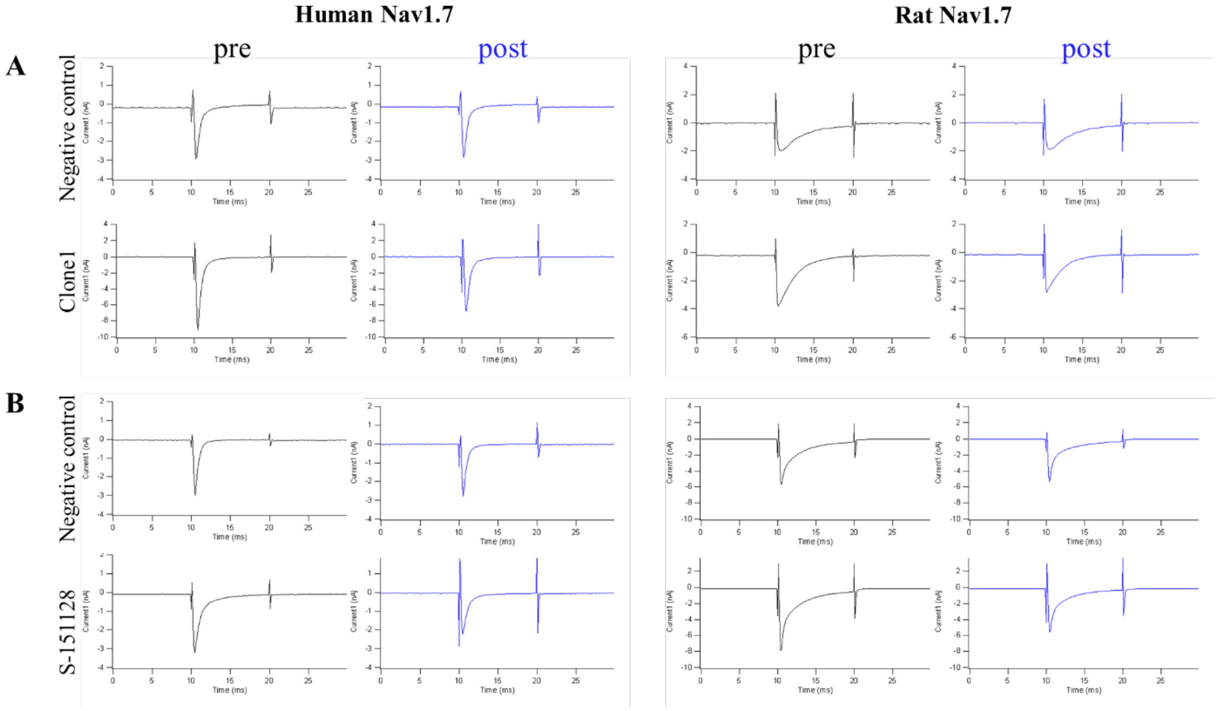

Figure S1. Representative traces of pre-post of sodium currents in HEK cells expressing human/rat Nav1.7

Whole-cell patch-clamp recordings were performed in HEK cells expressing human or rat Nav1.7 to evaluate the effects of antibody treatment on Nav1.7-mediated sodium currents. Representative trace of sodium currents were presented before and after application of either the negative control antibody or anti-Nav1.7 antibodies at a concentration of 100 µg/mL (A: Clone1, B S-151128).

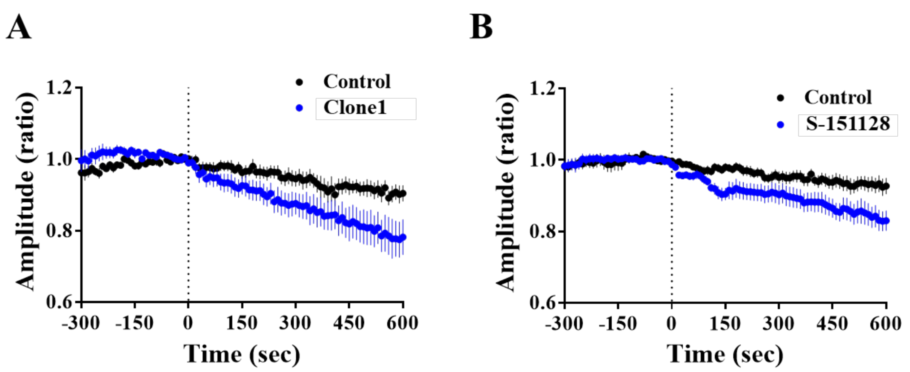

Figure S2. Time course of functional inhibition in rat Nav1.7

Whole cell patch clamp recordings were conducted on HEK cells expressing rat Nav1.7 to assess the inhibitory effects of antibodies at a concentration of 100 μg/mL on the sodium current. The data are presented as the mean ± SEM (n = 10 to 12). The time-course of peak currents during the experiments is shown in A and B, with the dotted lines indicating the start of antibody perfusion.

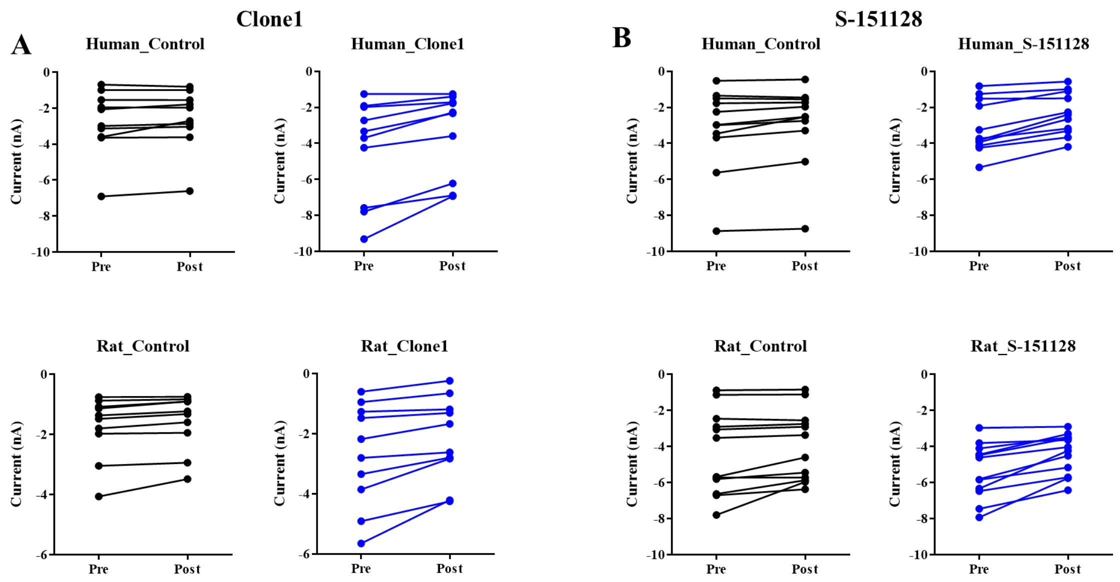

Figure S3. Pre-post changes of sodium currents in HEK cells expressing human/rat Nav1.7

Whole-cell patch-clamp recordings were performed in HEK cells expressing human or rat Nav1.7 to evaluate the effects of antibody treatment on Nav1.7-mediated sodium currents. Sodium currents were recorded before and after application of either the negative control antibody or anti-Nav1.7 antibodies at a concentration of 100 μg/mL. Sodium currents (nA) in each cell are shown for each treatment condition in the study of Fig. 3A-F.

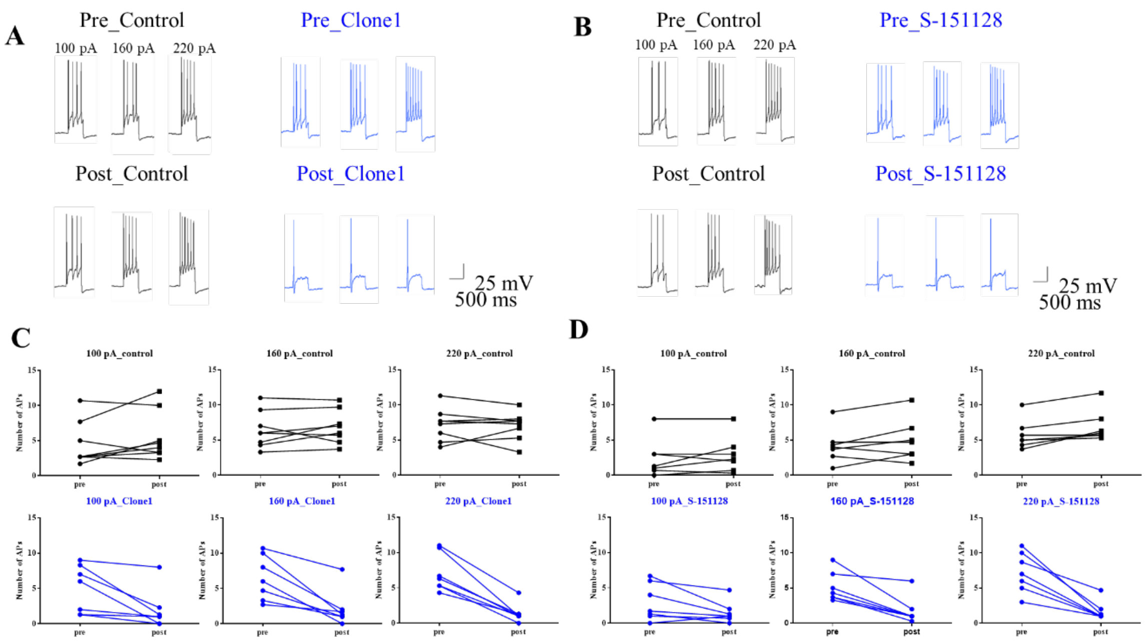

Figure S4. Representative membrane potential traces and pre–post changes in action potential firing in rat DRG neurons.

Membrane potential recordings were performed in visually identified rat dorsal root ganglion (DRG) neurons to assess action potential firing. Representative traces from a single neuron during current injections of 100, 160, and 220 pA are shown in (A, B), corresponding to the recordings presented in Fig. 3G–J. Pre–post changes in the number of APs before and after each treatment are plotted in (C, D).

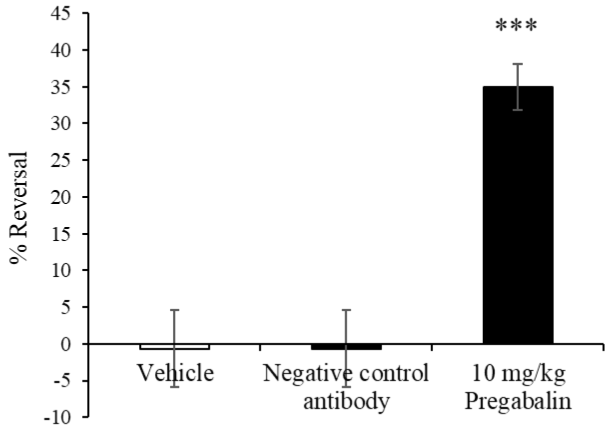

Figure S5. Evaluation of analgesic effect of negative control antibody

The analgesic effect of the negative control antibody (10 mg/kg) was assessed based on the paw withdrawal threshold (PWT). PWT was measured before treatment and at 5 h after a single administration of the antibody, or 3 h after pregabalin treatment. Data are shown as the mean ± SEM (n = 6 per group). Statistical significance was evaluated by two-way ANOVA followed by Tukey's post hoc test. ***$p < 0.001$ versus the vehicle-treated group.
